# Rehabilitation of Frail Older Adults after Hip Fracture Surgery: Predictors for the Length of Geriatric Rehabilitation Stay at a Skilled Nursing Home

**DOI:** 10.3390/jcm13154547

**Published:** 2024-08-03

**Authors:** Sanne M. Krakers, Sanne Woudsma, Dieuwke van Dartel, Marloes Vermeer, Miriam M. R. Vollenbroek-Hutten, Johannes H. Hegeman, on behalf of the Up&Go after a Hip Fracture Group

**Affiliations:** 1 Department of Trauma Surgery, Ziekenhuisgroep Twente, 7609 PP Almelo, The Netherlands; 2 Department of Biomedical Signals and Systems, University of Twente, 7500 AE Enschede, The Netherlands; 3 Geriatric Rehabilitation Department, ZorgAccent, 7442 KH Nijverdal, The Netherlands; 4 ZGT Academy, Ziekenhuisgroep Twente, 7609 PP Almelo, The Netherlands

**Keywords:** geriatric rehabilitation, hip fracture, length of stay, surgery, older patients, predictors

## Abstract

**Background:** Approximately 50% of older patients hospitalized for hip fractures are admitted to a geriatric rehabilitation department at a skilled nursing home. Given the wide variation in rehabilitation stay lengths, predicting the length of stay upon hospital discharge would help manage patients’ recovery expectations and create appropriate therapy schedules. Existing literature on length of stay predictors included both acute hospital and in-hospital rehabilitation phases or involved small sample sizes. The present study aims to identify predictors for the length of geriatric rehabilitation stay in skilled nursing homes for older patients after hip fracture surgery upon hospital discharge. **Methods:** This retrospective cohort study was conducted from 1 October 2017 to 1 July 2023, including 561 patients. Potential predictors of the length of geriatric rehabilitation stay were first tested univariately, with variables showing *p* < 0.15 entered into a multivariate forward linear regression model. **Results:** This model identified the following independent predictors of a longer length of geriatric rehabilitation stay: Functional Ambulation Categories (FACs) 0 (B = 29.9, 95% CI 24.1–35.7), 1 (B = 18.0, 95% CI 11.8–24.2), 2 (B = 12.0, 95% CI 7.1–17.0), or 3 (B = 3.6, 95% CI −1.2–9.4) at hospital discharge vs. FAC 4, living independently with home care services (B = 5.9, 95% CI 2.5–9.3) or in a residential home prior to the hip fracture (B = 0.2, 95% CI −7.4–7.8) vs. living independently without home care services, non- or partial weight-bearing mobilization vs. full weight-bearing mobilization (B = 15.4, 95% CI 8.5–22.2), internal fixation vs. hemiarthroplasty (B = 4.7, 95% CI 1.4–7.9), in-hospital delirium (B = 7.0, 95% CI 2.2–11.7), and in-hospital heart failure (B = 7.9, 95% CI 0.5–15.3). The explained variance was 32.0%. **Conclusions:** This study identified FAC at hospital discharge, premorbid living situation, postoperative weight-bearing protocol, surgery type, in-hospital delirium, and in-hospital heart failure as independent predictors of the length of geriatric rehabilitation stay. Future investigations are needed to identify additional predictors, such as cognitive functioning, to better predict the length of geriatric rehabilitation stay upon hospital discharge.

## 1. Introduction

After hip fracture surgery, approximately 50% of patients aged ≥70 years are admitted to a geriatric rehabilitation department at a skilled nursing home [1]. In 2017, the Department of Trauma Surgery at Ziekenhuisgroep Twente (ZGT), Almelo, the Netherlands, developed and implemented a multidisciplinary transmural care pathway in collaboration with three skilled nursing homes (TriviumMeulenbeltZorg, ZorgAccent, and Carintreggeland) in the vicinity of ZGT to synchronize the care processes and to gain more insights into the rehabilitation process of older patients after hip fracture surgery. In all skilled nursing homes, there was a “rehabilitation climate”, meaning that every daily activity was used as a therapeutic moment. Both caregivers and family members actively participated in the rehabilitation process. Multidisciplinary treatment was focused on the patient’s rehabilitation goals. The multidisciplinary team consisted of a nursing home physician, physiotherapist, occupational therapist, dietician, psychologist, and the nursing staff. For all geriatric rehabilitation departments, the received therapy by the physiotherapist was the same. However, the first results of the multidisciplinary transmural care pathway showed an interquartile range (IQR) of 25–50 days for the length of geriatric rehabilitation stay among patients [2].

Due to the wide variation in the length of stay in the geriatric rehabilitation facility, it is difficult for hospital healthcare professionals to prepare patients for what to expect during the rehabilitation period and for geriatric rehabilitation healthcare professionals to create appropriate therapy schedules. It is desirable to be able to predict the length of geriatric rehabilitation stay upon hospital discharge. This enables patients to manage their recovery expectations at the rehabilitation department of a skilled nursing home. Being aware of what to expect helps patients prepare for what is coming and set realistic goals. Furthermore, predicting the length of geriatric rehabilitation stay upon hospital discharge may help healthcare professionals identify the rehabilitation needs of each patient. For example, patients who are expected to recover rapidly may benefit from a more intensive therapy schedule, allowing for the most efficient organization of rehabilitation therapy. 

There is a great diversity in the literature concerning factors influencing the length of geriatric rehabilitation stay in older patients after hip fracture surgery [3,4,5]. Higher age, trochanteric and subtrochanteric fractures compared to intracapsular fractures, comorbidities (Parkinson’s disease, diabetes, and dementia), complications (wound infection, delirium, urinary tract infection, and pneumonia), independent gait at hospital discharge compared to dependent gait, living alone prior to the hip fracture compared to living together, using mobility aids prior to the hip fracture compared to being mobile without aids, and a lower Barthel score at geriatric rehabilitation admission were found as predictors for a longer length of geriatric rehabilitation stay [3,4,5]. However, these variables were found in studies with small sample sizes, were collected during both the hospital and rehabilitation phases, or were associated with the total length of hospital stay, including the acute hospital and in-hospital rehabilitation phases. It therefore remains unclear which factors influence the actual length of geriatric rehabilitation stay at a skilled nursing home upon discharge from the hospital. This lack of clarity currently renders predictions of the length of geriatric rehabilitation stay upon hospital discharge impossible. 

The aim of this study is to identify predictors for the length of geriatric rehabilitation stay at a skilled nursing home for older patients after hip fracture surgery upon hospital discharge, using data collected from the transmural care pathway.

## 2. Materials and Methods

### 2.1. Study Design

**Figure 1:**
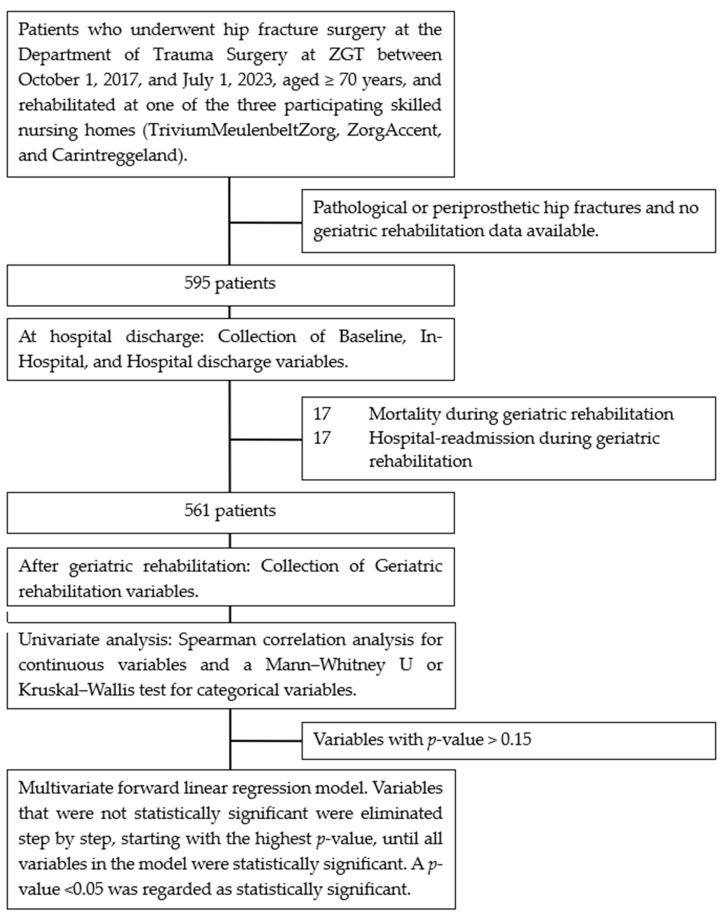
Flowchart of study design.

A retrospective cohort study was conducted from the first of October 2017 until the first of July 2023. Inclusion criteria for the study were patients aged ≥70 years who underwent hip fracture surgery (hemiarthroplasty or internal fixation) at the Department of Trauma Surgery at ZGT and rehabilitated at one of the three participating skilled nursing homes (TriviumMeulenbeltZorg, ZorgAccent, and Carintreggeland) (Figure 1). Exclusion criteria for this study were pathological or periprosthetic fractures or incomplete data collection of the rehabilitation process. 

The Medical Ethical Committee of Twente stated that this study did not require an assessment by the Medical Ethical Committee according to Dutch law. The study was approved by the Institutional Review Board of ZGT. All patients gave written informed consent to participate.

The primary outcome was the length of geriatric rehabilitation stay at the skilled nursing homes in days. For this study, baseline, in-hospital, hospital discharge, and geriatric rehabilitation variables were collected from the transmural care pathway database. Data were entered into Castor EDC, a cloud-based Electronic Data Capture platform (ISO 27001 certified) [6].

### 2.2. Baseline Variables

Age, gender, premorbid living situation, living alone versus together, availability of non-professional help, need for climbing stairs, Pre-Fracture Mobility Score (PFMS), premorbid Katz Index of Independence in Activities of Daily Living (Katz-ADL6), Charlson Comorbidity Index (CCI), American Society of Anesthesiologists physical status classification (ASA score), and Short Nutritional Assessment Questionnaire (SNAQ) were collected as baseline variables. 

The (P)FMS assesses the patients’ independence in walking and ranges from 1 (freely mobile without aids) to 5 (no functional mobility) [7]. The Katz-ADL6 assesses patients’ independence in ADL, where a score of 0 means fully independent in ADL and a score of 6 means fully dependent in ADL [8]. The CCI measures comorbidity, where a higher score means a higher comorbidity burden [9]. The ASA score measures the condition of a patient prior to surgery, with ASA 1–2 for a patient who has no or mild systematic diseases, ASA 3–4 for a patient who has severe systematic diseases, and ASA 5 for a moribund patient [10]. The SNAQ is a malnutrition screening tool, with SNAQ ≥ 2 indicating moderately malnourished patients and SNAQ ≥ 3 severely malnourished patients [11].

### 2.3. In-Hospital Variables

Hemoglobin (Hb) at hospital admission, hip fracture type, time until surgery, surgical treatment, postoperative weight-bearing protocol, and in-hospital complications were collected as in-hospital variables.

### 2.4. Hospital Discharge Variables

Functional Ambulation Categories (FACs) at hospital discharge, Fracture Mobility Score (FMS) at hospital discharge, Katz-ADL6 at hospital discharge, admission to which skilled nursing home (TriviumMeulenbeltZorg, Carintreggeland, or ZorgAccent), and the length of hospital stay were collected as hospital discharge variables. 

The FAC assesses the patients’ independence in walking and ranges from 0 (no functional mobility) to 5 (fully independent mobility) [7].

### 2.5. Geriatric Rehabilitation Variables

The hospital readmission rate and mortality rate were recorded as geriatric rehabilitation variables. The length of hospital might be influenced by logistical factors that cannot be predicted or influenced and, therefore, was excluded as a potential predictor of the primary outcome measure. 

### 2.6. Statistical Analysis

Continuous data were described as mean with standard deviation (SD) or as median with interquartile range (IQR) in case of a skewed distribution. Categorical data were presented as numbers with the corresponding percentage. Relationships between baseline, in-hospital, and hospital discharge variables and length of geriatric rehabilitation stay were first tested univariately using a Pearson or Spearman correlation analysis for continuous variables and an independent *t*-test, Mann–Whitney U test, ANOVA, or Kruskal–Wallis test for categorical variables. Variables with a *p*-value < 0.15 were entered into a multivariate forward linear regression model to ensure that no potential variables would be missed. Subsequently, variables that were not statistically significant were eliminated step by step, starting with the highest *p*-value, until all variables in the model were statistically significant. A *p*-value < 0.05 was regarded as statistically significant. All analyses were performed using SPSS version 22.0 (IBM Corp., Armonk, NY, USA).

## 3. Results

In the study period from the first of October 2017 until the first of July 2023, a total of 604 older patients were included. Of those patients, 9 had incomplete data (no geriatric rehabilitation data available) and were excluded, leaving 595 older patients in this study (Table 1).

### 3.1. Patient Characteristics

The mean (SD) age was 82.5 (7.8) years and 70.9% (*n* = 422) of the patients were female. Severe comorbidities (ASA ≥ 3) were observed in 68.2% (*n* = 406) of the patients. In 50.3% (*n* = 299), the hip fracture type was a femoral neck fracture and in 49.7% (*n* = 296), a trochanteric or subtrochanteric femur fracture. In 60.2% (*n* = 358) of the patients, the time until surgery was within 24 h and in 6.7% (*n* = 40) after 48 h. In 59.8% (*n* = 356), the surgical treatment was internal fixation (Proximal Femur Nail Antirotation (PFNA), Dynamic Hip Screw (DHS), or cannulated screws) and in 40.2% (*n* = 239), a hemiarthroplasty. After surgical treatment, 93.8% (*n* = 558) of the patients were allowed to put full weight on the operated leg. During the hospital stay, 47.4% (*n* = 282) of the patients had no complications, 20.2% (*n* = 120) were anemic and needed a blood transfusion, 12.4% (*n* = 74) had delirium, and 4.6% (*n* = 28) heart failure.

### 3.2. Living Situation and Functional Status

Prior to the hip fracture, 59.8% (*n* = 356) of the patients were living independently without home care services, 34.8% (*n* = 207) independently with home care services, and 4.9% (*n* = 29) were living in a residential home. Furthermore, 33.3% (*n* = 198) of the patients were living together with a relative and 85.5% (*n* = 509) could invoke non-professional help. Before the fracture, 38.3% (*n* = 228) of the patients were able to walk fully independently without aids (PFMS 1), 54.5% (*n* = 324) used a walking aid (PFMS 2) or aids (PFMS 3), and 6.2% (*n* = 37) were only able to walk independently indoors (PFMS 4). Most patients were independent in ADL before the fracture (median (IQR) premorbid Katz-ADL6 was 0 (0–2)). At hospital discharge, 15.6% (*n* = 93) of the older patients had no functional mobility (FAC 0), 69.0% (*n* = 410) needed assistance during walking (FAC 1–3), and 15.1% (*n* = 90) were independent in mobility (FAC 4–5). In addition, most patients were dependent in ADL at hospital discharge (median (IQR) Katz-ADL6 was 4 (4–5)).

### 3.3. Length of Hospital and Geriatric Rehabilitation Stay 

The median (IQR) length of hospital stay was 8 (6–10) days. The median (IQR) length of geriatric rehabilitation stay at the skilled nursing home was 35 (24–47) days. Furthermore, 17 (2.9%) patients died during the rehabilitation phase and 17 (2.9%) patients were readmitted to the hospital during geriatric rehabilitation stay.

### 3.4. Predictors for Length of Geriatric Rehabilitation Stay

For the univariate analysis, 34 (5.7%) patients were excluded, because of readmission to the hospital or death during a geriatric rehabilitation stay. Results from the univariate analysis are presented in Table 2. 

Although the length of geriatric rehabilitation stay was not normally distributed, the residual plot of the multivariate linear regression model did show a normal distribution. Therefore, the multivariate linear regression analysis was still the appropriate analysis to use. Age, premorbid living situation, the need for climbing stairs, PFMS, premorbid Katz-ADL6, ASA score, surgical treatment, postoperative weight-bearing protocol, in-hospital anemia, in-hospital heart failure, in-hospital delirium, uncomplicated hospital course, FAC at hospital discharge, and Katz-ADL6 at hospital discharge were included in the multivariate linear regression model. Because hip fracture type was strongly associated with surgical treatment, it was not included in the model. Surgical treatment was regarded to be more patient-specific, as it depends on both the type of fracture and the condition of the patient. FMS at hospital discharge was also not included in the model as it was strongly associated with FACs at hospital discharge. The FAC was considered more relevant in the transmural care pathway compared to the FMS because it is more widely used in geriatric rehabilitation departments to assess patient progress. Furthermore, the rehabilitation department of the skilled nursing homes was not included in the model due to its association with the FAC at hospital discharge. Skilled nursing home A had the highest percentage of patients with a lower FAC at hospital discharge compared to skilled nursing homes B and C within their respective populations. This was a coincidence, as the skilled nursing home placement procedure does not depend on the FAC. The FAC was considered more relevant compared to the rehabilitation department of skilled nursing homes because the FAC is clinically more relevant. 

The final multivariate model included the following variables: FAC at hospital discharge, premorbid living situation, postoperative weight-bearing protocol, surgical treatment, in-hospital delirium, and in-hospital heart failure (Table 2). FAC 0 (B = 29.9, 95% CI 24.1–35.7), FAC 1 (B = 18.0, 95% CI 11.8–24.2), FAC 2 (B = 12.0, 95% CI 7.1–17.0), or FAC 3 (B = 3.6, 95% CI −1.2–9.4) at hospital discharge versus (vs.) FAC 4, living independently with home care services (B = 5.9, 95% CI 2.5–9.3) or in a residential home prior to the hip fracture (B = 0.2, 95% CI −7.4–7.8) vs. living independently without home care services, non- or partial weight-bearing mobilization vs. full weight-bearing mobilization (B = 15.4, 95% CI 8.5–22.2), internal fixation vs. hemiarthroplasty (B = 4.7, 95% CI 1.4–7.9), in-hospital delirium (B = 7.0, 95% CI 2.2–11.7), and in-hospital heart failure (B = 7.9, 95% CI 0.5–15.3) were independent predictors of a longer geriatric rehabilitation stay. The explained variance of this model was 32.0%.

## 4. Discussion

Independent predictors for a longer length of geriatric rehabilitation stay were a lower FAC at hospital discharge compared to FAC 4; living independently with home care services prior to the hip fracture compared to living independently without home care services; non- or partial weight-bearing mobilization compared to full weight-bearing mobilization; internal fixation compared to hemiarthroplasty; in-hospital delirium; and in-hospital heart failure.

A substantial amount of research exists on the factors influencing the length of stay after hip fracture surgery. However, many studies focus on factors influencing the length of hospital stay [12,13,14,15] or the length of stay in other rehabilitation settings (e.g., private care rehabilitation, in-hospital rehabilitation, or both the acute hospital and rehabilitation phases) [3,12,13,14,15,16,17,18], making them difficult to compare with this study. Previously identified predictors for a longer length of geriatric rehabilitation stay include higher age [3,16,18]; trochanteric and subtrochanteric fractures versus intracapsular fractures [3]; complications (wound infection, delirium, urinary tract infection, and pneumonia) [3,16]; comorbidities (Parkinson’s disease, diabetes, and dementia) [3,16,18]; independent gait at hospital discharge versus dependent gait [4]; living alone prior to the hip fracture versus living together [5,18]; using mobility aids prior to the hip fracture versus being mobile without aids [5,17]; a lower Barthel score at geriatric rehabilitation admission [5]; a lower Functional Independence Measure score at rehabilitation admission [16]; a lower Abbreviated Mental Test Score [17]; and a higher pain score at rehabilitation admission [18]. Among all these previously identified predictors, this study confirmed that in-hospital delirium is a predictor for a longer length of geriatric rehabilitation stay. Additionally, the previously identified predictor of trochanteric and subtrochanteric fractures versus intracapsular fractures can be compared to the predictor found in this study: internal fixation versus hemiarthroplasty. This is because the type of hip fracture is strongly associated with the choice of surgical treatment. However, this study did not include hip fracture type in the multivariate model, as surgical treatment was considered to be more patient-specific. Furthermore, the previously identified predictor of independent gait at hospital discharge versus dependent gait can be compared to a predictor found in this study: lower FAC at hospital discharge compared to FAC 4. This is because the FAC score also assesses the degree of walking independence of the patient. The remaining previously identified predictors for a longer length of geriatric rehabilitation stay do not align with the predictors found in this study. A possible explanation for this difference is that other studies used small study populations or different rehabilitation settings. The present study utilized a study population of 561 patients, specifically focusing on the length of stay in geriatric rehabilitation departments at skilled nursing homes. Consequently, this study solely examined in-hospital variables and did not include variables from the rehabilitation phase. This decision was made to prioritize the identification of predictors for the length of geriatric rehabilitation stay upon hospital discharge. Using variables from the rehabilitation phase does not allow for the early prediction of the length of geriatric rehabilitation stay. This hinders healthcare professionals from creating appropriate therapy schedules and preparing patients for what to expect during the rehabilitation period. Another explanation may be that this study assessed the comorbidity burden using the CCI, rather than assessing the specific types of comorbidities as possible predictors. This means that from this study, it is not clear if specific comorbidities may be predictors for the length of geriatric rehabilitation stay. However, this study chose to use the CCI because it captures not only the presence of comorbidities but also their severity, which may be more comprehensive than considering individual comorbidities alone [9]. 

Each identified independent predictor contributes to a longer length of geriatric rehabilitation stay in specific ways. For patients with a lower FAC at hospital discharge, functional self-reliance may be achieved more slowly, resulting in a longer length of geriatric rehabilitation stay. This highlights the importance of early mobilization during the hospital phase, as it enhances the functional recovery of patients [19,20,21]. Patients who lived independently with home care services prior to the hip fracture may require more complex care due to their decreased resilience. Although home care services offer some level of support, they may not fully address their rehabilitation needs following hip fracture surgery. As a result, these patients may require a longer length of geriatric rehabilitation stay. Receiving home care services following geriatric rehabilitation for patients who lived independently without home care services prior to the hip fracture may provide sufficient support and assistance to shorten the length of geriatric rehabilitation stay. Patients who lived in a residential home prior to the hip fracture may have sufficient support and assistance available at the residential home to shorten the length of geriatric rehabilitation stay. Patients with non- or partial weight-bearing mobilization are unable to mobilize or have a limited ability to do so and because of this situation receive a lower frequency of therapy during the first six weeks of rehabilitation. This results in a longer length of geriatric rehabilitation stay because their progress in regaining mobility and independence is slower. This is consistent with previous studies that have identified a relationship between a reduced length of hospital stay following hip fracture surgery and full weight-bearing mobilization [22,23]. Patients who undergo internal fixation may experience more post-operative pain and may require longer periods of non- or partial weight-bearing mobilization compared to those who undergo hemiarthroplasty [24,25]. This is due to the nature of the procedures: Hemiarthroplasty involves replacing the fractured hip with a prosthetic implant, whereas internal fixation involves stabilizing the fractured hip using intramedullary nails, plates, and/or screws. In-hospital delirium is characterized by symptoms such as cognitive impairment. Patients who develop an in-hospital delirium are less likely to mobilize after hip fracture surgery [26], which requires more time-intensive medical and nursing care. Similarly, patients with in-hospital heart failure face limitations during rehabilitation due to symptoms such as fatigue, reduced exercise capacity, dyspnea, and hypotension [14], also necessitating more time-intensive medical and nursing care. These findings are consistent with previous studies that have identified relationships between an extended length of hospital stay following hip fractures and either heart failure or delirium [14,27,28,29].

The results of this study are the first step towards a more efficient rehabilitation pathway. The length of geriatric rehabilitation stay can be better predicted upon hospital discharge, enabling patients to be better prepared for what to expect during the rehabilitation period. Furthermore, this information makes it possible to identify patients who may rehabilitate more quickly. This group may benefit from a more intensive therapy schedule to facilitate even faster recovery. Since early mobilization during the hospital phase enhances the functional recovery of patients, increasing the intensity of therapy early in postoperative rehabilitation may also contribute to faster recovery among other groups [19,20,21]. Shorter geriatric rehabilitation stays can address potential future capacity issues, as a faster patient flow will enable the treatment of an increasing number of patients with hip fractures.

The limitation of our study is the lack of information on cognitive functioning prior to admission to the geriatric rehabilitation department. No tests measuring cognitive functioning were conducted during the hospital phase. The presence of an in-hospital delirium is the only cognitive functioning status we assessed. However, cognitive functioning may significantly influence the length of geriatric rehabilitation stay, as patients with cognitive impairments may have difficulty performing physiotherapy exercises. A strength of our study lies in the inclusion of a great diversity of possible predictors from both the physical and social domains in the analyses of predictors for the length of geriatric rehabilitation stay. Furthermore, our results statistically confirm the independent predictors for a prolonged length of geriatric rehabilitation stay at skilled nursing homes. These predictors were previously thought to influence the length of stay based on clinical experience but lacked statistical proof. In addition, this study distinguishes itself by focusing exclusively on in-hospital variables, allowing for the early prediction of the length of geriatric rehabilitation stay. Lastly, this study is also valuable for developing a final prediction model in which the length of geriatric rehabilitation stay can be predicted at the time of hospital discharge; something that cannot be achieved with clinical experience alone. 

The explained variance of 32.0% in the multivariate model showed that other predictors for length of rehabilitation stay exist that were not included in the present study. Therefore, future research is recommended to optimize the multivariate model. Additional factors that are considered important in predicting the length of geriatric rehabilitation stay should be incorporated. Since early mobilization and physical activity enhance the functional recovery of patients [19,20,21], continuously monitored physical activity using a sensor during the in-hospital phase is relevant to add to the model. Other relevant factors to add to the model are cognitive functioning assessed with a cognitive screening test during the hospital phase and the fear of falling. Fear of falling could influence the length of geriatric rehabilitation stay, as it may impede rehabilitation by being associated with anxiety and self-efficacy [30]. The occurrence of complications during the rehabilitation stay could also influence the length of the rehabilitation stay [3]. However, this factor cannot be included in the analysis to predict the length of rehabilitation stay upon hospital discharge. 

## 5. Conclusions

A lower FAC at hospital discharge vs. FAC 4, living independently with home care services prior to the hip fracture vs. living independently without home care services, non- or partial weight-bearing mobilization vs. full weight-bearing mobilization, internal fixation vs. hemiarthroplasty, in-hospital delirium, and in-hospital heart failure were found as independent predictors of a longer length of geriatric rehabilitation stay. These results are the first step towards a more efficient rehabilitation pathway. However, with an explained variance of 32.0% in the multivariate model, there are more predictors of a longer length of stay that were not included in this study. Future investigations will be needed to identify additional predictors to better predict the length of geriatric rehabilitation stay upon hospital discharge. The ability to predict the length of geriatric rehabilitation stay more precisely will help healthcare professionals prepare patients for what to expect during the rehabilitation period and create appropriate therapy schedules, aiming for faster recovery. This may address potential future capacity issues, as a faster patient flow will enable the treatment of an increasing number of patients with hip fractures.

## Figures and Tables

**Table 1:** Patient characteristics.

		Total (*n* = 595)
Baseline variables		
Age in years; mean (SD)		82.5 (7.8)
Female gender; *n* (%)		422 (70.9)
Premorbid living situation; *n* (%)	Independent without home care services	365 (59.8)
	Independent with home care services	207 (34.8)
	Residential home	29 (4.9)
Living together; *n* (%)		198 (38.3)
Availability non-professional help; *n* (%)		509 (85.5)
Need for climbing stairs; *n* (%)		155 (26.1)
PFMS; *n* (%)	1—Freely mobile without aids	228 (38.3)
	2—Mobile outdoors with one aid	60 (10.1)
	3—Mobile outdoors with two aids or frame	264 (44.4)
	4—Some indoor mobility but never goes outside without help	37 (6.2)
	5—No functional mobility	0 (0)
Premorbid Katz-ADL6; median (IQR)		0 (0–2)
CCI; median (IQR)		1 (0–2)
ASA score; *n* (%)	ASA 1–2	184 (30.9)
	ASA 3–4	406 (68.2)
	ASA 5	0 (0.0)
SNAQ; median (IQR)		0 (0–0)
In-hospital variables		
Hb at hospital admission; median (IQR)		8.5 (7.0–9.0)
Hip fracture type; *n* (%)	Femoral neck	299 (50.3)
	Trochanteric/subtrochanteric femur	296 (49.7)
Time until surgery; *n* (%)	0–24 h	358 (60.2)
	24–36 h	139 (23.4)
	36–48 h	58 (9.7)
	> 48 h	40 (6.7)
Surgical treatment; *n* (%)	Internal fixation: PFNA, DHS, cannulated screws	356 (59.8)
	Hemiarthroplasty	239 (40.2)
Postoperative weight-bearing protocol; *n* (%)	Full weight-bearing	558 (93.8)
	Partial/non-weight-bearing	37 (6.2)
In-hospital complications; *n* (%)	Anemia	120 (20.2)
	Heart failure	28 (4.6)
	Delirium	74 (12.4)
	Pneumonia	57 (9.6)
	Pressure ulcers	29 (4.9)
	Urinary tract infection	36 (6.1)
	Wound infection	3 (0.5)
	Uncomplicated	282 (47.4)
Hospital discharge variables		
FACs at hospital discharge; *n* (%)	0—No functional mobility	93 (15.6)
	1—Dependent in mobility level 2	67 (11.3)
	2—Dependent in mobility level 1	167 (28.1)
	3—Independent mobility under supervision	176 (29.6)
	4—Independent mobility on a flat surface	90 (15.1)
FMS at hospital discharge; *n* (%)	1—Freely mobile without aids	0 (0)
	2—Mobile outdoors with one aid	0 (0)
	3—Mobile outdoors with two aids or frame	28 (4.7)
	4—Some indoor mobility but never goes outside without help	462 (77.6)
	5—No functional mobility	105 (17.6)
Katz-ADL6 at hospital discharge; median (IQR)		4 (4–5)
Rehabilitation department skilled nursing home; *n* (%)	Skilled nursing home A	304 (51.1)
	Skilled nursing home B	215 (36.1)
	Skilled nursing home C	76 (12.8)
Length of hospital stay in days; median (IQR)		8 (6–10)
Geriatric rehabilitation variables		
Hospital-readmission during rehabilitation; *n* (%)		17 (2.9)
Mortality during rehabilitation; *n* (%)		17 (2.9)
Length of geriatric rehabilitation stay in days; median (IQR)		35 (24–47)

SD, standard deviation; n, number; (P)FMS, (Pre-)Fracture Mobility Score; Katz-ADL, Katz Index of Independence in Activities of Daily Living; IQR, interquartile range; CCI, Charlson Comorbidity Index; ASA, American Society of Anesthesiologists physical status classification; SNAQ, Short Nutritional Assessment Questionnaire; Hb, hemoglobin; PFNA, Proximal Femoral Nail Antirotation; DHS, Dynamic Hip Screw; FACs, Functional Ambulation Categories.

**Table 2:** Univariate analysis and multivariate linear regression analysis factors length of geriatric rehabilitation stay.

		Univariate Analysis * (*n* = 561)	Multivariate Linear Regression Analysis (*n* = 561)	
		*p*	B (95% CI)	*p*
Baseline variables				
Age		0.020		
Female gender		0.713		
Premorbid living situation	Independent without home care services	<0.001	ref.	
	Independent with home care services		5.9 (2.5–9.3)	<0.001
	Residential home		0.2 (−7.4–7.8)	0.952
Living together		0.267		
Availability non-professional help		0.501		
Need for climbing stairs		0.127		
PFMS	1—Freely mobile without aids	<0.001		
	2—Mobile outdoors with one aid			
	3—Mobile outdoors with two aids or frame			
	4—Some indoor mobility but never goes outside without help			
	5—No functional mobility			
Premorbid Katz-ADL6		<0.001		
CCI		0.871		
ASA score	ASA 1–2	0.003		
	ASA 3–4			
SNAQ		0.983		
In-hospital variables				
Hb hospital admission		0.667		
Hip fracture type	Femoral neck	0.025		
	Trochanteric/subtrochanteric femur			
Time until surgery	0–24 h	0.405		
	24–36 h			
	36–48 h			
	>48 h			
Surgical treatment	Internal fixation: PFNA, DHS, cannulated screws	<0.001	4.7 (1.4–7.9)	0.005
	Hemiarthroplasty		ref.	
Postoperative weight-bearing protocol	Full weight-bearing		ref.	
	Partial/non-weight-bearing	<0.001	15.4 (8.5–22.2)	<0.001
In-hospital complications	Anemia	0.008		
	Heart failure	0.020	7.9 (0.5–15.3)	0.037
	Delirium	<0.001	7.0 (2.2–11.7)	0.004
	Pneumonia	0.871		
	Pressure ulcers	0.268		
	Urinary tract infection	0.348		
	Wound infection	0.260		
	Uncomplicated	<0.001		
Hospital discharge variables				
FAC at hospital discharge	0—No functional mobility	<0.001	29.9 (24.1–35.7)	<0.001
	1—Dependent in mobility level 2		18.0 (11.8–24.2)	<0.001
	2—Dependent in mobility level 1		12.0 (7.1–17.0)	<0.001
	3—Independent mobility under supervision		3.6 (−1.2–8.5)	0.141
	4—Independent mobility on a flat surface		ref.	
FMS at hospital discharge	1—Freely mobile without aids	0.014		
	2—Mobile outdoors with one aid			
	3—Mobile outdoors with two aids or frame			
	4—Some indoor mobility but never goes outside without help			
	5—No functional mobility			
Katz-ADL6 at hospital discharge		<0.001		
Rehabilitation department skilled nursing home	Skilled nursing home A			
	Skilled nursing home B	<0.001		
	Skilled nursing home C			

* Univariate analysis was performed using a Spearman correlation analysis for continuous variables and a Mann–Whitney U or Kruskal–Wallis test for categorical variables. *p*, *p*-value; ref., reference; (P)FMS, (Pre-)Fracture Mobility Score; Katz-ADL, Katz Index of Independence in Activities of Daily Living; CCI, Charlson Comorbidity Index; ASA, American Society of Anesthesiologists physical status classification; SNAQ, Short Nutritional Assessment Questionnaire; Hb, hemoglobin; PFNA, Proximal Femoral Nail Antirotation; DHS, Dynamic Hip Screw; FAC, Functional Ambulation Categories.

## Data Availability

Data are unavailable due to privacy restrictions.
